# Deficit mulched drip irrigation improved yield and quality while reduced water consumption of *isatis indigotica* in a cold and arid environment

**DOI:** 10.3389/fpls.2022.1013131

**Published:** 2022-09-30

**Authors:** Chenli Zhou, Hengjia Zhang, Fuqiang Li, Yong Wang, Yucai Wang, Zeyi Wang

**Affiliations:** College of Water Conservancy and Hydropower Engineering, Gansu Agricultural University, Lanzhou, China

**Keywords:** deficit irrigation, *isatis indigotica*, water consumption characteristics, yield, water use efficiency, quality

## Abstract

Deficit irrigation is an effective alternative to traditional irrigation, as it improves crop productivity and conserves water. However, crops may be sensitive to deficit irrigation-induced water stress at different periods. To access the effect of deficit irrigation on the growth, water consumption characteristics, yield, and quality of *Isatis indigotica* (woad), we performed a three-year (2017-2019) mulched drip irrigation field experiment. Woad plants were provided adequate water supply at the seedling stage but were subjected to mild (65–75% field water capacity FC), moderate (55–65% FC), and severe (45–55% FC) water deficit at the vegetative growth, fleshy root growth and fleshy root maturity stages, respectively; plants supplied with adequate water throughout the growth period served as a control (CK, 75–85% FC). The water consumption characteristics, agronomic traits, dry matter content and distribution, yield, and quality of these plants were measured at various growth stages. The results showed that the total water consumption in water deficit was significantly less than that in CK by 4.44–10.21% (*P*< 0.05). The dry matter content of plants treated with moderate (WT2 and WT5) and severe (WT3) water deficit was reduced by 12.83–28.75%. The economic yield of mild water deficit-treated plants was higher during vegetative growth (WT1) and fleshy root growth (WT4), while the water use efficiency of these plants was significantly increased by 7.84% and 6.92% at the two growth stages, respectively. Continuous mild water deficit (WT4) enhanced the contents of indigo, indirubin, (R,S)-goitrin, polysaccharides, and soluble proteins during vegetative growth and fleshy root growth, while moderate and severe water deficit were detrimental to the quality of woad plants. Thus, continuous mild water deficit during vegetative and fleshy root growth periods (WT4) is optimal for the cultivation of woad in the cold and cool irrigation district of the Hexi Oasis region.

## 1 Introduction

The imbalance between crop production and agricultural water supply critically inhibits agricultural production globally. Unfortunately, climate change, global warming, population growth, urbanization, and industrial development are increasingly exacerbating water shortage, which threatens agricultural development and food security around the world (Mckenzie and Williams, 2015; Yunusa et al., 2018). Currently, more than 50% of the agricultural land worldwide is facing drought (Geng et al., 2016), which has been a major factor constraining agricultural production (Pelling et al., 2004). To ensure sustainable agricultural production while also to supply the world’s growing need for food, gradually improving soil and water productivity is critical. Currently, agriculture accounts for about 70% of the total global water use, most of which is utilized for crop irrigation (Kang et al., 2017). Irrigation is a fundamental requirement for agricultural production in arid and semi-arid areas. However, water scarcity is the primary factor that inhibits agricultural development in these regions. Therefore, the establishment of more rational irrigation strategies, such as changing the irrigation systems and decreasing the frequency or volume of irrigation, is needed to manage agricultural production and irrigation more efficiently in arid and semi-arid regions.

China is a huge agricultural economy that supports about one quarter of the global population, based on the widespread use of irrigation and fertilization (Zong et al., 2021). The inland arid regions of central and western China have been under serious drought stress for a long time caused by extremely low precipitation and high evaporation (Meng et al., 2013). Hence, new irrigation strategies need to be established that will enable more efficient use of the finite water resources in these areas. Excessive irrigation can lead to crop lodging or reduced tolerance to waterlogging (saturated soils), which can inhibit crop growth, resulting in lower yields and wasteful use of water resources. Managing the amount of irrigation is essential for achieving superior yields and biomass accumulation. Deficit irrigation is a worthwhile and profitable production trend for arid areas where water scarcity limits crop production (Liu et al., 2006; Shahnazari et al., 2007; Geerts and Raes, 2009). Deficit irrigation aims to enhance crop water use efficiency (WUE) by decreasing the amount or frequency of irrigation. Compared with adequate irrigation, deficit irrigation can conserve water, decrease production costs, and increase water productivity, in turn creating a balance between yield and water inputs (Shahzad et al., 2017; Cui et al., 2020). Overall, the environmental influence of crops can be diminished. However, not all the growth stages of crops are susceptible to water stress because of insufficient irrigation. Irrigation schedules are, therefore, developed based on the knowledge of the water deficit sensitivity of crops at different growth stages; water requirements of the crops are met during the water deficit sensitive stages, and water deficit is applied at the insensitive stages (Win et al., 2014). Large number of studies have found that deficit irrigation can reduce water use while keeping sufficient yields and improving fruit quality (Favati et al., 2009; Patane et al., 2011; Kusçu et al., 2014; Giuliani et al., 2016).

*Isatis indigotica* (woad) is a traditional herbal medicine. The leaves of woad (Da Qing Ye) plants has been used to treat type B encephalitis, mumps, and influenza viruses (Han et al., 2011). The root of woad (Ban Lan Gen) plants can lower body temperature and relieve sore throat, and has been used in the treatment of hepatitis, mumps, influenza, mononucleosis, and other diseases (Zhang D. D. et al., 2019). It is one of the drugs prescribed for the anticipation and treatment of the fatal severe acute respiratory syndrome (SARS) in China (Ke et al., 2012). Minle, which is situated in the Hexi Corridor, is one of the most vital herbal cultivation areas in Northwest China, owing to its unique climatic conditions. The cultivation of Chinese herbs in this region plays an essential role in safeguarding the growth of authentic medicinal herbs. However, development of the woad industry has long been severely constrained by local water scarcity due to low rainfall, extremely high evaporation, and deep groundwater burial. To resolve the challenge of water shortage, mulched drip irrigation, which is considered as an effective water conservation technology, has been widely used for crop cultivation in this region. Previous studies showed that a suitable regulated deficit irrigation treatment could reduce the irrigation amount and crop evapotranspiration (ETc) and enhance WUE and crop quality without significantly affecting crop yield (Carbonell-Barrachina et al., 2015; Cano-Lamadrid et al., 2015; Chai et al., 2016; Lahoz et al., 2016). However, achieving high-level accumulation of phytochemicals and biomass in crop plants simultaneously is difficult under any environmental conditions (Bumgarner et al., 2012), and an higher yields are usually accompanied by lower quality (Graham et al., 2018). Moreover, the research by agronomists or nutritionists on agricultural water consumption, crop productivity, quality, and human health are generally isolated (Nyathi et al., 2018). Therefore, a combination of regulated deficit irrigation and mulched drip irrigation is essential to comparatively study the response of water deficit in the growth dynamics, yield, and quality of woad. Well-formed data are needed to establish the optimum level and timing of water deficit required for achieving high yield and quality, and to understand the growth variables of woad and the distribution of dry matter among different plant structures. We hypothesized that mild water deficit would facilitate yield enhancement and quality improvement for woad. The main aims of this study were as follows: 1) to investigate the effects of regulated deficit mulched drip irrigation on the growth, water consumption regularity, yield, and quality of woad in the Hexi region; and 2) to establish the optimal degree and duration of deficit irrigation, which could be used as a theoretical guide for the water-saving and efficient cultivation of woad in the cold and arid regions of China.

## 2 Materials and methods

### 2.1 Study site description

**Figure 1:**
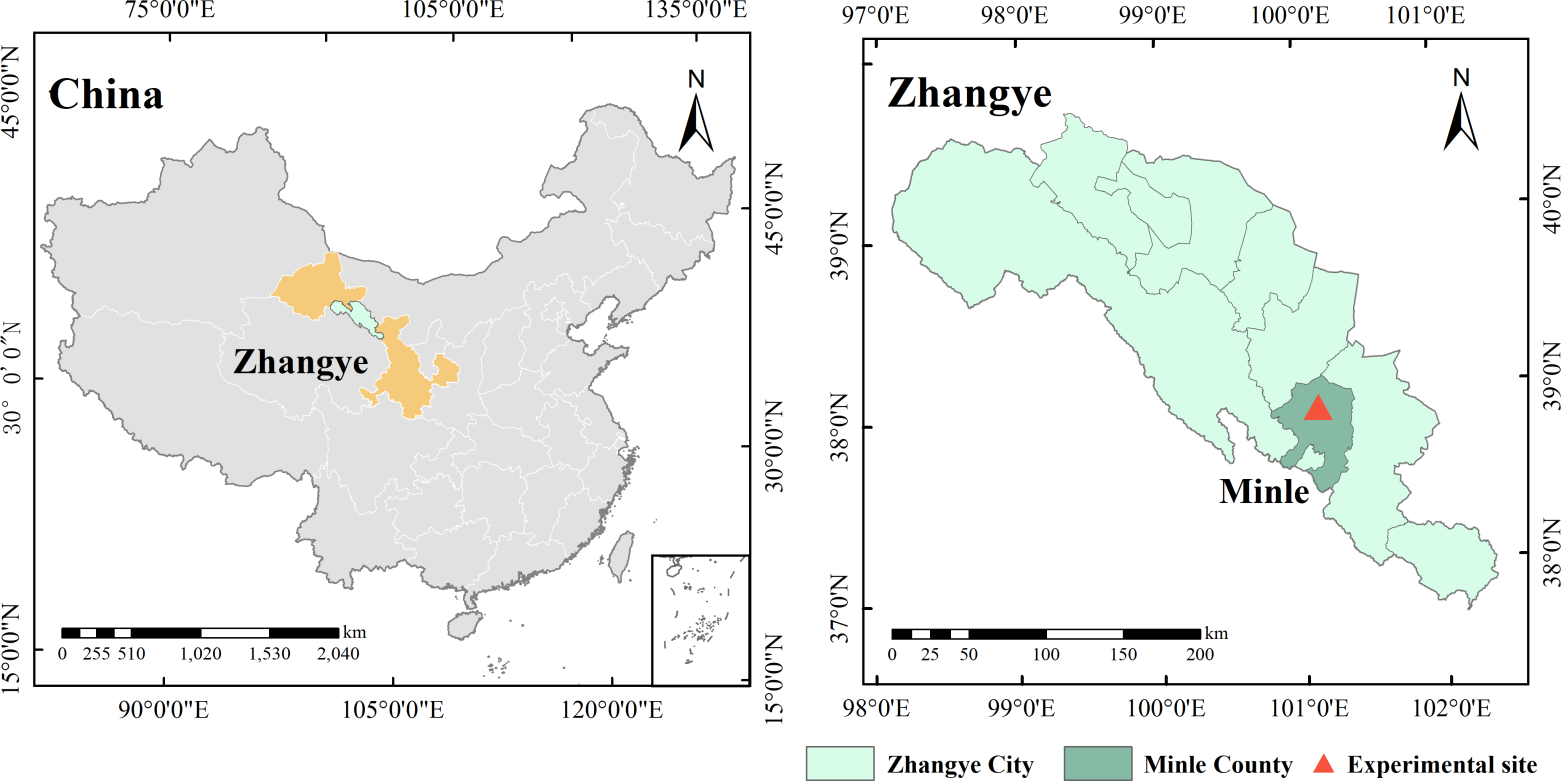
Location of field experiments (Minle, China).

Field experiments were conducted from 2017 to 2019 at Yimin Irrigation Experiment Station, Minle County of Gansu Province, China (38°39′′N, 100°43′′E, and 1,970 m asl). The experimental site is located in the middle of the Hexi Corridor of Gansu Province, China (**Figure 1**), and has a typical continental desert-steppe climate with an average annual temperature of 7.6 °C, dryness of 5.85, average annual sunshine duration of 2,932 h, frost-free stage of approximately 150 d, average annual evaporation of 1,638 mm, and average annual precipitation of only 183–345 mm, which mainly happens from June to September. Over the last 30 years (1986–2016), the average monthly temperature and precipitation were the highest in July and August, respectively. The agricultural soil type is light loam, with soil bulk density of approximately 1.46 g·cm^-3^ in the 0–100-cm layer, and organic matter, alkali-hydrolyzable nitrogen, available potassium, and available phosphorus contents of 12.4 g·kg^-1^, 57.3 mg·kg^-1^, 191.7 mg·kg^-1^, and 15.9 mg·kg^-1^, respectively, in the 0–20-cm layer. According to the test data from the experiment station, the field water capacity and permanent wilting point of soil at 100-cm depth were 24.0% and 7.1%, respectively.

**Figure 2:**
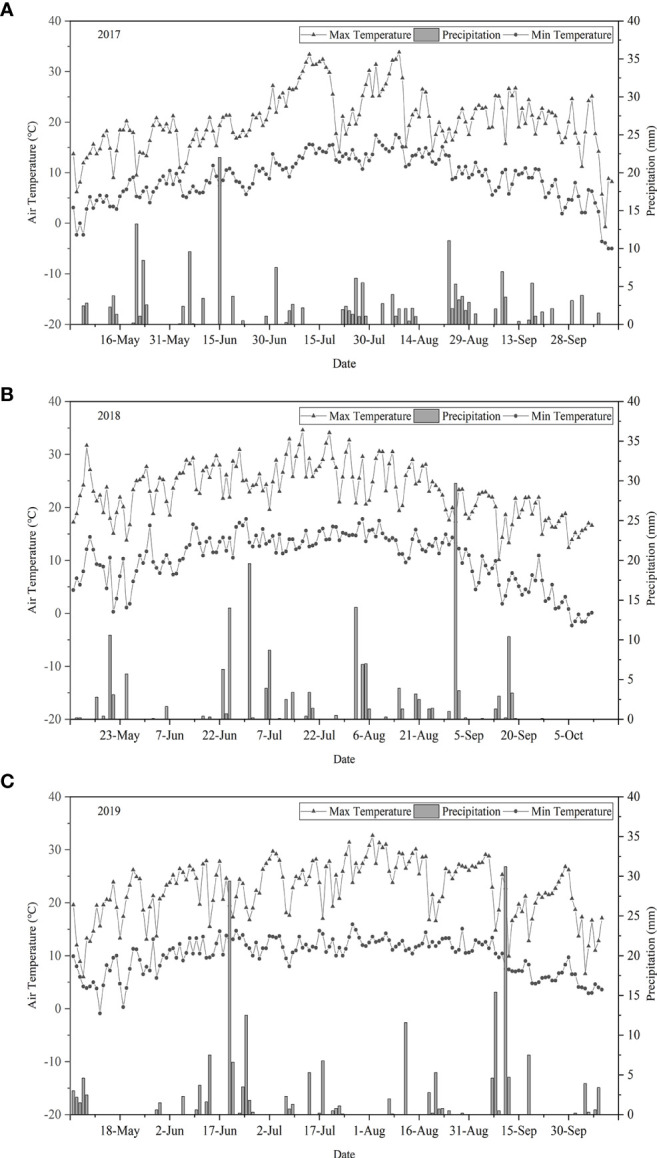
Daily maximum and minimum temperature and precipitation data during the growth period of *I. indigotica* in 2017 **(A)**, 2018 **(B)**, and 2019 **(C)**.

**Figure 2** shows the temperature and precipitation patterns in the growth stage of *I. indigotica* (woad). Temperature varied in a boundary throughout the growth stage, and the variation trends were similar each year. In 2017, 2018, and 2019, the average daily maximum temperature was 25.7°C, 24.6°C, and 23.5°C respectively, and the average daily minimum was 2.7°C, 3.9°C, and 5.1°C respectively. The effective rainfall throughout the growth period was 196.5, 210.3, and 191.3 mm in 2017, 2018, and 2019, respectively. Precipitation in 2017 was mostly distributed within the periods of fleshy root growth and fleshy root maturity. High precipitation events were observed mainly during vegetative growth and fleshy root growth in 2018 and 2019, with rainfall amount exceeding 15 mm in each event.

### 2.2 Experimental design and field management

**Figure 3:**
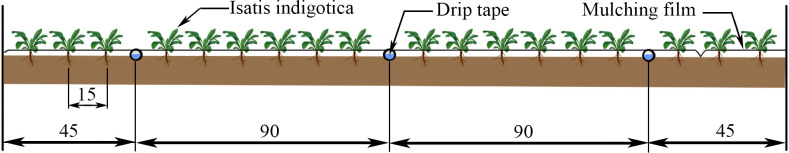
Layout of the experimental design mulched drip irrigation. The numbers unit is cm.

The experiment was conducted as a single factor randomized block trial. Before sowing, the experimental field was prepared by mechanically plowing the land to a depth of 30 cm, manually removing the weeds, leveling the land, applying the base fertilizer, and laying the drip irrigation belt. The strip spacing, drip head spacing, and average drip head flow were 90 cm, 30 cm, and 2.4 L·h^-1^, respectively (**Figure 3**). The drip irrigation belt was overlaid with a 120-cm wide colorless plastic film. The varieties selected for the experiment were the main local varieties (*Isatis indigotica Fort.*). The growth period of woad was divided into four stages: seedling, vegetative growth, fleshy root growth, and fleshy root maturity. Four gradients of soil moisture were set: adequate water supply (75–85% of the field water capacity [FC]), mild water deficit (65–75% of FC), moderate water deficit (55–65% of FC), and severe water deficit (45–55% of FC). Six regulated water deficit treatments and one control (CK) treatment were established in each of the three growing seasons of 2017, 2018, and 2019, with three replications in each treatment. Details of the different water treatments are summarized in **Table 1**. The area of each plot was 13.5 m^2^ (2.7 m × 5 m). Irrigation was conducted using drip irrigation along with plastic film mulching, and the irrigation amount was measured using water meters. To prevent lateral infiltration of soil moisture between plots, a 60-cm wide plastic film was used to separate the adjacent plots from each other. The planting density of woad was 8.3 × 10^5^ plants·ha^-1^. The main agronomic management practices are described in **Table 2**.

**Table 1:** Experimental design.

Treatments	Seeding	Vegetative growth	Fleshy root growth	Flesh root maturity
WT1	75–85% ^a^	65–75%	75–85%	75–85%
WT2	75–85%	55–65%	75–85%	75–85%
WT3	75–85%	45–55%	75–85%	75–85%
WT4	75–85%	65–75%	65–75%	75–85%
WT5	75–85%	55–65%	55–65%	75–85%
WT6	75–85%	65–75%	75–85%	65–75%
CK	75–85%	75–85%	75–85%	75–85%

a Percentages indicate the amount of water supplied relative to the field capacity (FC).

**Table 2:** Agronomic practices employed for *I. indigotica* cultivation in experimental field plots at Minle of Gansu Province, China.

Agronomic practice	2017	2018	2019
Planting density	8.3 × 10^5^ plants·ha^−1^		
Fertilizer	220, 330, and 120 kg·ha^-1^ of urea, calcium superphosphate, and potassium, respectively		
Planting pattern	Full film cover and flat crop planting		
Mulching time	May 1	May 8	May 3
Sowing time	May 2	May 9	May 4
Sowing method	Hole sowing		
Sowing depth	5 cm	5 cm	5 cm
Planting row spacing	15 cm	15 cm	15 cm
Planting spacing	8 cm	8 cm	8 cm
Harvesting time	October 11	October 12	October 10
Field management	Manual weed removal and disease and insect control		

### 2.3 Measurements and calculations

#### 2.3.1 Soil water content

Soil water content was determined using the drying method. At randomly selected spots in each experimental plot, soil samples were collected to a depth of 100 cm within six graded soil layers (0–10, 10–20, 20–40, 40–60, 60–80, and 80–100 cm) using an auger. Since the root activity range of woad spans a depth of 0–45 cm, the average soil water in the 0–60-cm layer was considered as the soil water content within the planned soil wetting depth, namely the basis of irrigation, and the change in soil moisture content within the 0–100-cm layer was considered as the basis of crop water consumption calculation. Soil moisture was measured once before woad sowing and every 10 days after seedling emergence, and additional measurements were recorded after rainfall and irrigation.

Soil water content was calculated using the following equation (Zhang et al., 2019):


$$\theta_{j}=\frac{m_{j1}-m_{j2}}{m_{j2}}\times 100\%$$

where *θ_j_* is the soil water content of layer *j* (%); *m_j1_* is the weight of the wet soil layer *j* (g), and *m_j2_* is the weight of dry soil layer *j* (g).

Irrigation amount was calculated using the following formula (Zhang et al., 2019):


$$M=10\gamma HP(\theta_{1}-\theta_{2})$$

where M is the irrigation amount (mm); γ is the soil bulk density (g·cm^-3^); H is the planned wetting layer depth (60 cm); P is the designed wetting ratio under drip irrigation (%); θ_1_ is the upper limit of soil moisture content (%) in the experimental design; and θ_2_ is the actual measured soil moisture content (%).

#### 2.3.2 Soil water storage

Soil water storage was calculated according to the following equation (Liu et al., 2018):


V=γ×h×ω×10

where v is the soil water storage capacity (mm); γ is the measured soil bulk density (g·cm^-3^); h is the thickness of the soil layer (cm); ω is the soil water content (%).

#### 2.3.3 Water consumption

The water consumption of woad plants was calculated using the water balance method (Saleh et al., 2008), according to the following equation:


$$ET=10\sum_{i=1}^{n}\gamma_{i}H_{i}(W_{i1}-W_{i2})+M+P+K-C$$

where *ET* is the water consumption of woad plants at a certain growth stage (mm); *i* is the soil layer number; *H_i_* is the thickness of the *i^th^* layer (cm); *γ_i_* is the soil bulk density of the *i^th^* layer (1.45 g·cm^-3^); *W_i1_* and *W_i2_* are the soil mass water contents (%) of the *i^th^* layer at the beginning and end, respectively, within a measurement period; *P* is the rainfall amount within a certain time (mm); K is the amount of water replenished from the groundwater into the 0–100-cm soil layer (mm); and C is the amount of water lost as deep seepage (mm). The value of *K* was assumed as 0, since the test area had a deep water table with no deep water replenishment from the groundwater. Additionally, since the planned wetting layer was 60 cm, there was no deep seepage water existed; therefore, the value of *C* was considered as 0.

#### 2.3.4 Growth indicators

Three uniformly growing woad plants were selected from each plot at each growth stage, and all growth indexes were measured separately. The height and taproot length of woad of plants were measured using a steel tape with an accuracy of 0.1 cm. The taproot diameter was measured using Vernier calipers with an accuracy of 0.02 mm.

#### 2.3.5 Dry matter content

Three plants of uniform size were selected from every plot at different growth period. The sampled plants were rinsed with water several times, and soaked dry with filter paper. Then, the aerial part of each plant (stems and leaves) was separated from the belowground part (roots), both of which were then dried and separately weighed (Zhang et al., 2019).

#### 2.3.6 Yield

Upon reaching maturity, woad plants were excavated from a unit area (1 m^2^) in each plot, and the total yield of plants per hectare was calculated.

#### 2.3.7 Wue

The WUE (kg·ha^-1^·mm^-1^) of woad was calculated using the following equation (Zhang et al., 2011):


$$WUE=\frac{Y}{ET}$$

where, Y is the yield (kg·ha^-1^); and ET is the total water consumption (mm).

#### 2.3.8 Quality

The contents of active ingredients, including indigo, indirubin, and (R,S)-goitrin, in *I. indigotica* roots were determined by high-performance liquid chromatography (HPLC), as described in the Chinese Pharmacopoeia (Wang Y. C. et al., 2021). The sample was placed at 105 °C for 15 min, then dried at 60 °C to a constant mass, crushed and passed through a 60-mesh sieve. The powder was used for the determination of the contents of indigo, indirubin, and (R,S)-goitrin. Chromatographic conditions: Shimadzu LC-20 HPLC, SPD-20 detector; Column temperature 40 °C; the mobile phase were V (methanol) ∶V (water) =75∶25 (indigo and indirubin), and V (methanol) ∶V (0.02% phosphoric acid solution) =10∶90 ((R,S)-goitrin). The detection temperature was 25 °C; The detection wavelengths were 289 nm (indigo and indirubin) and 245 nm ((R,S)-goitrin).

Indigo and indirubin: Weighed 0.5 g of sample powder for each treatment group, added 100 mL of trichloromethane, soaked for 15 h and then extracted by reflux in a Soxhlet extractor at 80°C in a water bath until colorless, and the extract was concentrated and evaporated under reduced pressure, The residue was dissolved in methanol and the volume was fixed to 100 m L. After shaking, the residue was filtered by 0.45 μm microporous membrane. (R,S)-goitrin: Weighed 1 g of the sample powder of each treatment group, added 50 mL of distilled water and weighed the total mass; decocted for 2 h and weighed the total mass again, and make up the lost mass with distilled water; shaked well and filter, centrifuged the filtrate, the supernatant was the sample solution.

Sample solution 20 μL was absorbed and analyzed by HPLC according to the above chromatographic analysis conditions, and the contents of indigo, indirubin, and (R,S)-goitrin in the sample were calculated according to the standard curve, respectively.

The contents of polysaccharides and soluble proteins in woad roots were determined using phenol-concentrated sulfuric acid and Kaumas Brilliant Blue G-250, respectively (Wang Y. C. et al., 2021).

### 2.4 Data analysis

Statistical analysis of the soil moisture content of woad plots and the water consumption characteristics, height, taproot length, dry matter accumulation, yield, quality, and economic benefits of plants was conducted using the IBM SPSS Statistics 23.0 software. The data were performed to one-way analysis of variance (ANOVA), and the differences between treatments were analyzed using the Tukey’s least significant difference (LSD) test at a 5% level. Utilizing Origin 2019 for plotting and Microsoft Excel 2013 for data processing. Data analysis were performed using the three-year average.

## 3 Results

### 3.1 Crop water consumption

#### 3.1.1 Soil water storage

**Figure 4:**
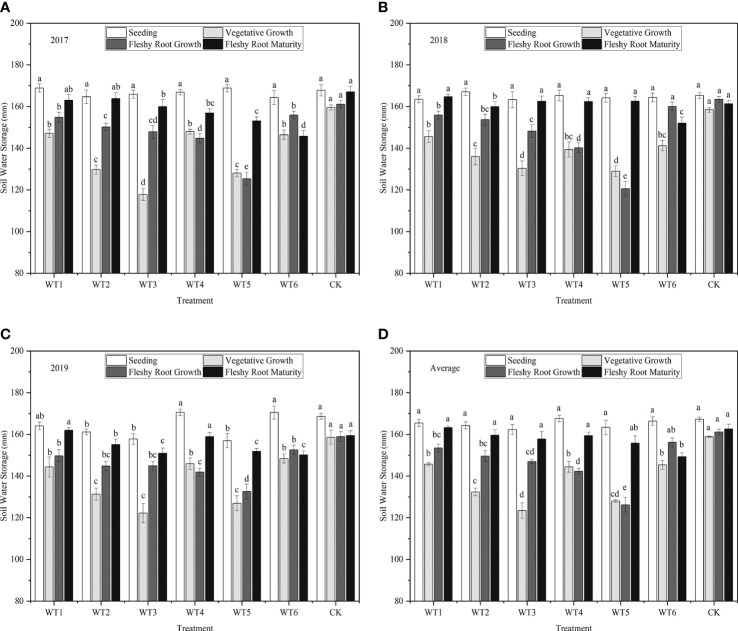
Soil water storage under different treatments. **(A–D)** describe the soil water storage in 2017, 2018,2019 and the average of these three years, respectively. Different lowercase letters in the same column indicate significant differences (*P*< 0.05). Data were presented as mean ± SE (n = 3).

**Figure 4** shows the amount of water stored in in the soil at 0–60 cm depth at different growth stages of *I. indigotica* (woad) in 2017, 2018, and 2019. In all treatments, crops were subjected to sufficient water supply at seedling, and soil water storage in the 0–60 cm layer ranged from 162.32 to 167.58 mm. Soil water storage was the highest (158.88 mm) in the control (CK) during the vegetative growth period, and this value was 8.29–22.29% higher than those in all water deficit treatments. No significant difference was observed in soil water storage among WT1, WT4, and WT6 treatments (*P* > 0.05) and between WT2 and WT5. Additionally, the WT1, WT2, and WT3 treatments showed no significant differences in soil water storage during the fleshy root growth period but displayed significantly (*P*< 0.05) reduced soil water storage than the CK (by 4.76%, 7.17%, and 8.79%, respectively). In the fleshy root maturity, WT1 showed the highest soil water storage (163.23 mm), and WT1, WT2, WT3, WT4, and CK had no significant differences in soil water storage; however, soil water storage in WT6 was significantly less that than in the CK by 8.19%.

#### 3.1.2 Water consumption

##### 3.1.2.1 Water consumption at each growth stage

**Figure 5:**
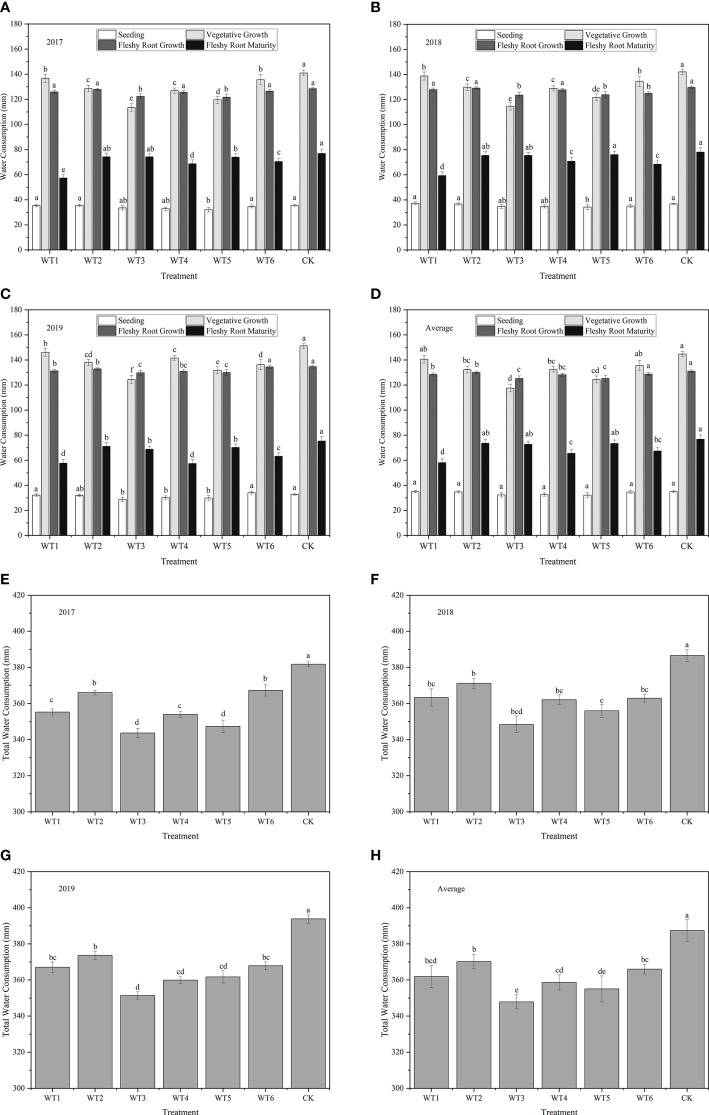
Water consumption and total water consumption of *I. indigotica* plants in different treatments. **(A–D)** describe the water consumption in 2017, 2018, 2019, and the average of these three years, respectively, while **(E–H)** describe the total water consumption in 2017, 2018, 2019, and the average of these three years, respectively. Different lowercase letters in the same column indicate significant differences (*P*< 0.05). Data were presented as mean ± SE (n = 3).

The dynamics of woad water consumption at each growth stage in the three experimental years is shown in **Figure 5**. The water consumption of woad showed a single peak curve, with a rise followed by a decline. In each water deficit treatment, water consumption increased from the seedling to the vegetative growth stage, with the highest water consumption occurring at the vegetative growth and fleshy root growth, followed by a decline during fleshy root maturity, while the biggest increase in water consumption appeared during vegetative growth. Water consumption at the seedling stage was the lowest (32.04–35.01 mm), which accounted for 9.02–9.04% of the total water consumption. Therefore, the vegetative growth and fleshy root growth were the most water demanding stages of woad. The stems and leaves of woad plants grew rapidly, as evident from the speedy increase in leaf area, and the leaves gradually covered the ground during vegetative growth and fleshy root growth. Additionally, transpiration, root system growth, and taproot length and diameter increased greatly, resulting in the highest water consumption during vegetative growth and fleshy root growth. The highest water consumption (144.75 mm) was observed in CK during vegetative growth, while the lowest water consumption was detected in WT3 plants, which was significantly less than that of CK by 18.80%. After entering the fleshy root growth phase, water consumption in both CK and WT6 was at the same level (*P* > 0.05) and was significantly higher than that in all other treatments. Water consumption during fleshy root maturity was significantly less (*P*< 0.05) than that during fleshy root growth, which ranged from 58.09–76.72 mm only.

##### 3.1.2.2 Total water consumption

Total water consumption over the three years was the highest in CK (387.38 mm), and the corresponding values in the other treatments were significantly lower by 4.44–10.21% (*P*< 0.05) (**Figure 5**). Among all water deficit treatments, WT3 showed the lowest water consumption (351.41 mm) throughout the growth period, which was significantly less than the water consumption in CK by 10.21%. Water consumption in WT5 was significantly less than that in WT2 and CK by 4.10% and 8.35%, respectively, revealing that continuous water deficit at a moderate level during the fleshy root growth period could significantly reduce total water consumption. No significant difference (*P* > 0.05) in total water consumption was observed among the WT1, WT4, and WT6 treatments, and water consumption in these treatments was significantly less than that in CK by 6.58%, 7.42%, and 5.51%, respectively. Additionally, mild water deficit significantly reduced water consumption during vegetative growth but had no significant effect on water consumption during fleshy root growth and fleshy root maturity.

##### 3.1.2.3 Water consumption intensity at each growth stage

**Figure 6:**
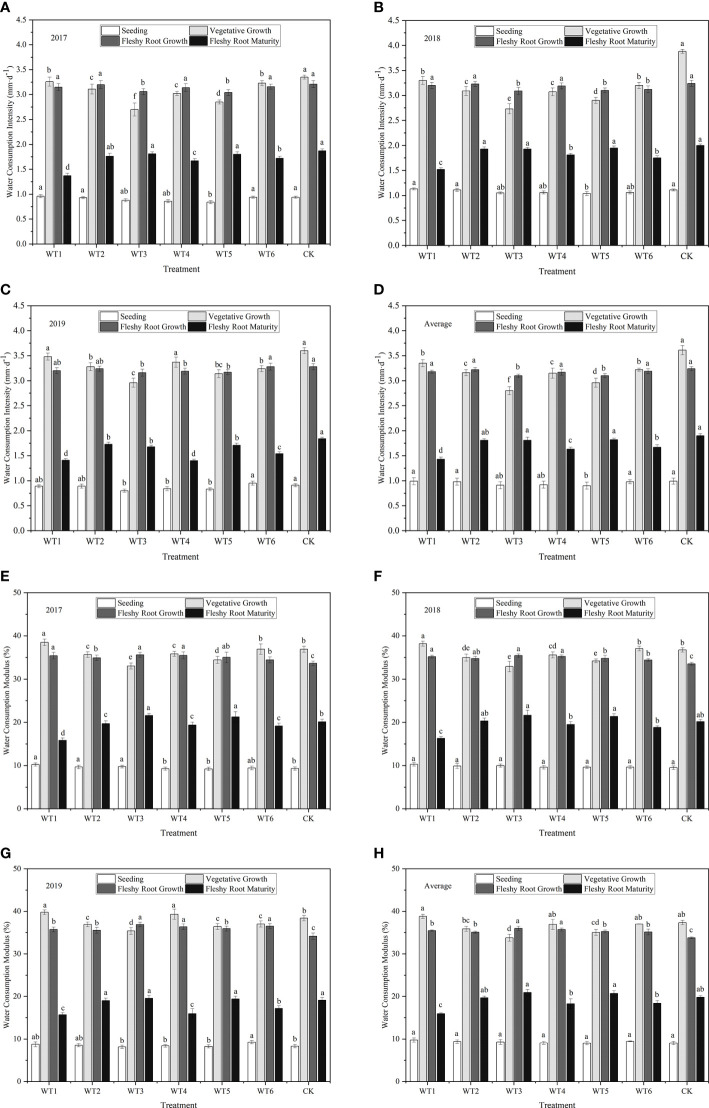
Water consumption intensity and water consumption modulus of *I. indigotica* plants in different water deficit treatments. **(A–D)** describe the water consumption intensity in 2017, 2018, 2019, and the average of these three years, respectively, while **(E–H)** describe the water consumption modulus in 2017, 2018, 2019, and the average of these three years, respectively. Different lowercase letters in the same column indicate significant differences (*P*< 0.05). Data were presented as mean ± SE (n = 3).

The daily water consumption intensity of woad in each water deficit treatment showed a single peak curve during the growing season (**Figure 6**). Water consumption intensity was lowest at the seedling and highest at vegetative and fleshy root growth stages, followed by a continuous decline during fleshy root maturity. The greatest increase in daily water consumption intensity was observed during vegetative growth. The daily water consumption intensity was the close (*P* > 0.05) among all treatments without water deficit treatment at the seedling stage, which was the lowest (0.91–0.99 mm·d^-1^) compared with other growth stages. In each water deficit treatment, the daily water consumption intensity was significantly (*P*< 0.05) higher at the vegetative growth than that at the seedling. The highest daily water consumption intensity was 3.61 mm·d^-1^ in CK under adequate water supply, and no significant difference was found between WT1 and CK. Compared with CK, the plant daily water consumption intensity in WT2 and WT5 treatments was significantly decreased by 12.47% and 18.01%, respectively, and that in WT3 was significantly decreased by 22.44%. This indicates that mild water deficit does not significantly decrease the daily water consumption intensity of woad, while moderate and severe water deficit seriously reduce the daily water consumption intensity. At the fleshy root growth stage, the woad plants grew rapidly and consumed larger amounts of water under conditions of rising temperature, leading to a daily water consumption intensity of approximately 3.17 mm·d^-1^. In addition, the daily water consumption intensity in WT1, WT2, WT4, and WT6 treatments showed no significant difference compared with CK, while that in WT3 and WT5 was significantly reduced by 4.32% and 4.32% respectively. The daily water consumption intensity of woad plants at the fleshy root maturity stage (1.43–1.90 mm·d^-1^) was significantly lower than that during fleshy root growth. The CK treatment showed the highest daily water consumption intensity (1.90 mm·d^-1^), which was significantly higher than that observed in the other treatments. On the other hand, plants in the WT1 treatment showed the lowest daily water consumption intensity (1.43 mm·d^-1^) at vegetative growth, which was significantly less than that in CK by 24.74%.

##### 3.1.2.4 Water consumption modulus

The water consumption modulus of woad in each water deficit treatment also showed a single peak curve during the growing season, with the following order of peak intensity: seedling< fleshy root maturity< vegetative growth and fleshy root growth (**Figure 6**). No significant difference (*P* > 0.05) in water consumption modulus was observed among the different treatments at the seedling stage, with the lowest water consumption modulus of only 9.03–9.76%. WT1 showed the lowest average water consumption modulus (15.96%), which was significantly (*P*< 0.05) lower than that of CK by 19.43%. The average water consumption modulus of woad in WT2, WT3, and WT5 was not significantly different from that in CK, with the highest value in WT3 (20.93%). The average water consumption modulus of woad during the vegetative and fleshy root growth periods was significantly greater than that during other growth periods, with mean values of 36.40% and 35.21%, respectively. The average water consumption modulus during the vegetative growth period increased by 3.93% in WT1 and significantly decreased by 9.58% and 6.24% in WT3 and WT5, respectively, compared with CK. This illustrated that mild water deficit did not significantly reduce the water consumption modulus of woad, while moderate and severe water deficit significantly reduced the water consumption modulus. In WT3 and WT5, the average water consumption modulus of woad increased by 35.99% and 35.26%, respectively, during fleshy root growth than that during vegetative growth.

### 3.2 Crop growth and dry matter accumulation

#### 3.2.1 Final plant height, taproot length, and taproot diameter

**Figure 7:**
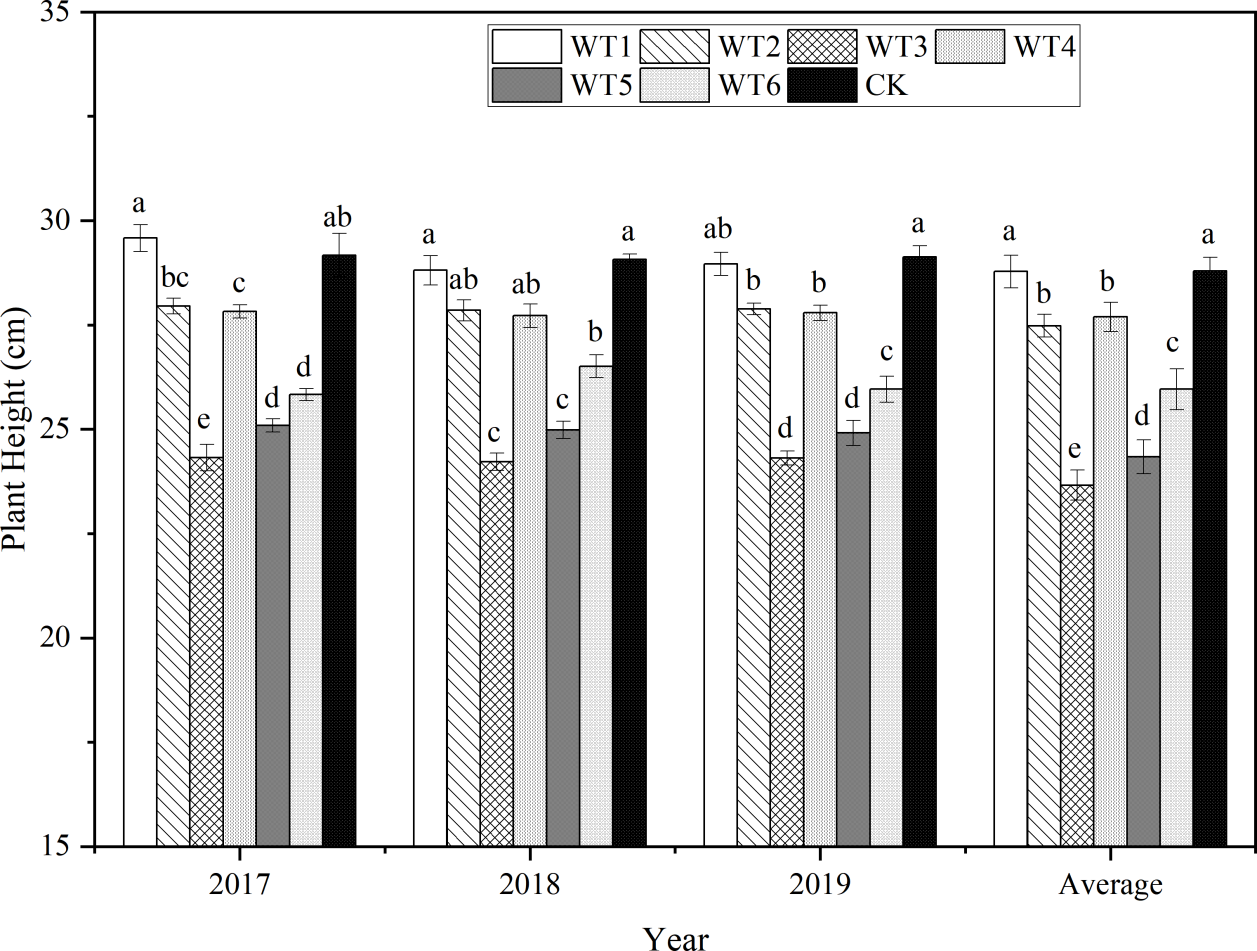
Final height of *I. indigotica* plants in different water deficit treatments. Different lowercase letters in the same column indicate significant differences (*P*< 0.05). Data were presented as mean ± SE (n = 3).

At the end of the growth period, woad plants were the tallest (final plant height: 29.12 cm) in CK (**Figure 7**), and plants in WT1 and CK showed no significant difference in the final height. Compared with CK, the final plant heights in other water deficit were significantly less (*P*< 0.05) by 4.22–16.62%, while that in WT2 was significantly reduced by 4.22%. The final plant height was significantly reduced by 10.40% in WT5 compared with WT2, and that in WT3 was the lowest (24.28 cm), significantly lower than the final plant height in CK by 16.62%. Therefore, both moderate and severe water deficits severely reduced the final plant height of woad. Compared with CK and WT4, the final plant height in WT6 was significantly reduced by 10.37% and 6.05%, respectively, revealed that mild water deficit during fleshy root growth and fleshy root maturity significantly diminished the final plant height of woad. Accordingly, the final plant height of woad was not only impacted by the degree of water deficit but also by the water deficit stage.

**Figure 8:**
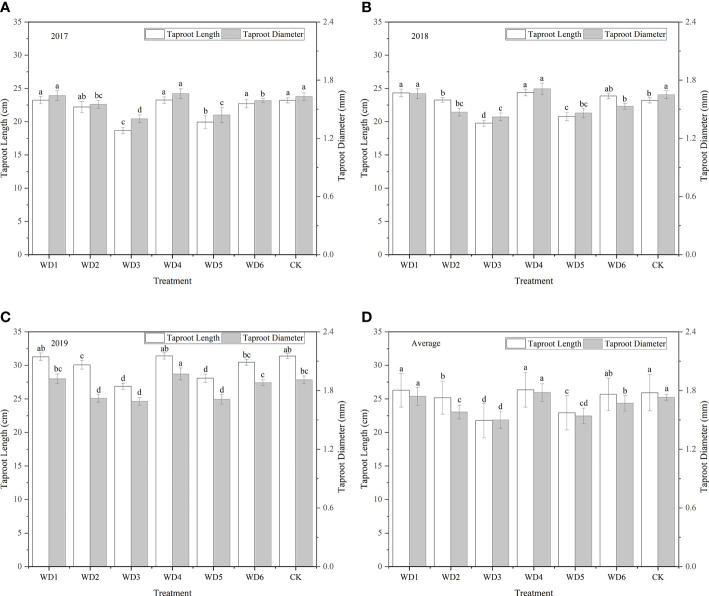
Taproot length and diameter of *I. indigotica* plants in different water deficit treatments. **(A–D)** describe the taproot length and diameter in 2017, 2018, 2019, and the average of these three years, respectively. Different lowercase letters in the same column indicate significant differences (*P*< 0.05). Data were presented as mean ± SE (n = 3).

The final taproot length (26.35 cm) and taproot diameter (1.78 cm) were the highest in WT4 (**Figure 8**) but showed no significant differences compared with the corresponding values in WT1 and CK (*P* > 0.05). This suggests that mild water deficit did not cause obvious degradation of the taproot length and diameter of woad plants; instead, mild water deficit was advantageous to the growth of the plant root system. The final taproot length and taproot diameter were reduced by 1.00–16.04% and 3.47–13.29%, respectively, in other water deficit treatments (WT2, WT3, WT5, and WT6) compared with CK. Compared with CK, The final taproot length and diameter were significantly reduced by 2.89% and 8.67%, respectively, in WT2, and by 16.04% and 13.29%, respectively, in WT3. This illustrates that both moderate and severe water deficits significantly decreased the final taproot length and taproot diameter of woad plants, and the rate of reduction in both these parameters increased with the increase in deficit degree.

#### 3.2.2 Dry matter accumulation

**Figure 9:**
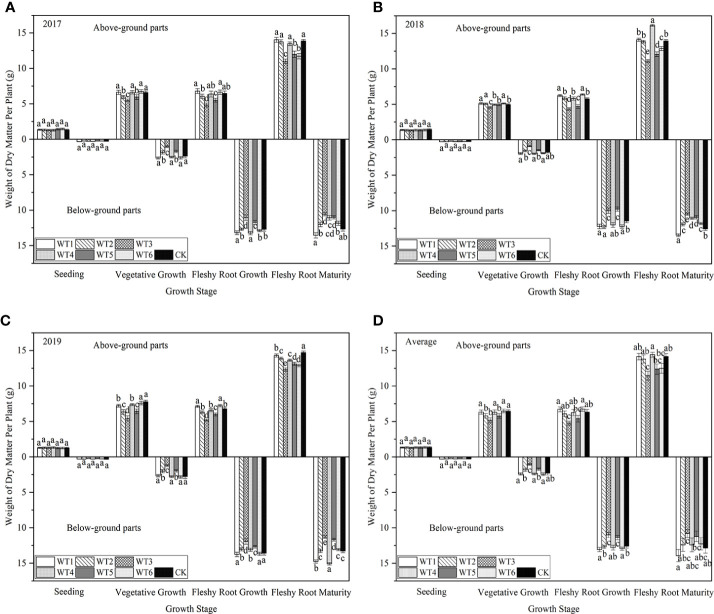
Dry matter accumulation in *I. indigotica* plants in different water deficit treatments. **(A–D)** describe the dry matter accumulation in 2017, 2018, 2019, and the average of these three years, respectively. Different lowercase letters in the same column indicate significant differences (*P*< 0.05). Data were presented as mean ± SE (n = 3).

The dry matter accumulation rate of woad presented an S-shaped variation pattern throughout the growing season (**Figure 9**). The total dry matter accumulation gradually increased as the growth period of woad progressed, reaching a maximum at fleshy root maturity. The total dry matter accumulation in WT1, WT4, and WT6 showed no significant difference (*P* > 0.05) from that in CK during the vegetative growth period, whereas the dry matter accumulation was significantly decreased (*P*< 0.05) by 12.83%, 28.75%, and 14.20% in WT2, WT3, and WT5, respectively, compared with CK. During the fleshy root growth period, the total dry matter accumulation was the highest in WT1 (19.78 g), which was similar to that in WT6 and CK. At the fleshy root maturity stage, the highest total dry matter accumulation was observed in WT1. Although the total dry matter accumulation in WT1 was not significantly different from that in WT4 and CK, it was significantly higher than that in WT6 by 11.86%, illustrating that mild water deficit at fleshy root maturity decreases dry matter accumulation.

### 3.3 Yield and WUE

**Figure 10:**
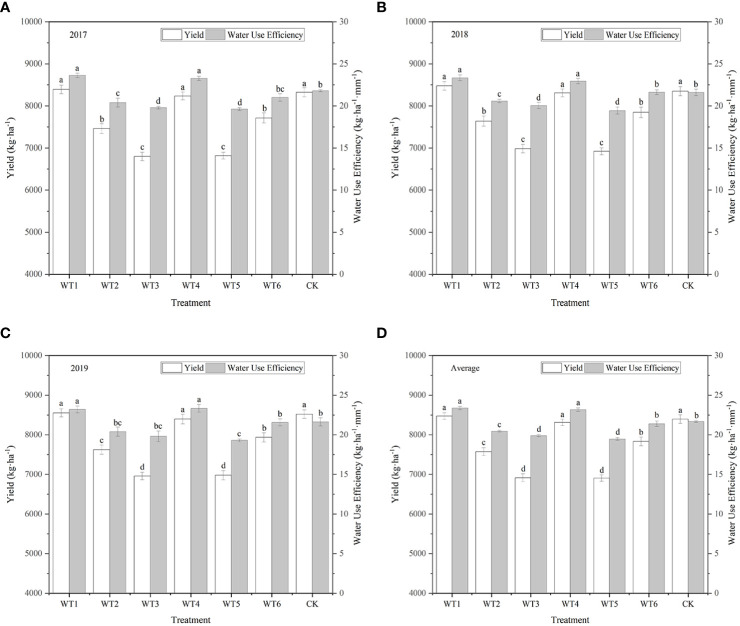
Yield and WUE of *I. indigotica* plants in different water deficit treatments. **(A–D)** describe the yield and WUE in 2017, 2018, 2019, and the average of these three years, respectively. Different lowercase letters in the same column indicate significant differences (*P*< 0.05). Data were presented as mean ± SE (n = 3).

The economic yield of woad was higher in WT1 and WT4 than in CK, although the difference was not significant (**Figure 10**), indicating that mild water deficit during vegetative growth and fleshy root growth periods did not significantly affect the economic yield of woad. Economic yield in WT6 was significantly reduced by 6.74% compared with CK, indicating that mild water deficit during the vegetative growth and fleshy root maturity periods negatively impacts the economic yield of woad. The economic yield of woad was significantly reduced by 9.80–17.74% in the other water deficit treatments compared with CK, indicating that moderate and severe water deficits resulted in a significant reduction in the yield of woad, which increases with the degree of water deficit.

Compared to CK, WUE in WT1 and WT4 was significantly improved by 7.84% and 6.92%, respectively (**Figure 10**). WUE in WT6 was lower than that in CK, although the difference was not significant. This demonstrates that mild water deficit does not significantly reduce WUE. The WUE of woad in the other treatments was significantly reduced by 5.63–10.24% compared with CK, indicating that moderate and severe water deficits lead to a obvious reduction in WUE of woad. The WUE in WT6 was significantly lower than that in WT4 by 7.68%, showing that the WUE of woad is also influenced by the plant growth stage.

### 3.4 Economic benefits

Improvement in the efficiency of mulched drip irrigation of woad was mainly achieved by reducing input costs and increasing economic yield. Regulated deficit irrigation helps conserve irrigation water, while film mulching improves the moisture content and temperature of farmland soil, which reduces weed growth, pest infestation, and production input cost. As shown in **Table 3**, the production cost, yield, and output value of woad plants in each water deficit treatment were determined, and the net income and revenue increase rate were calculated. The cost of water input was 0.22 Renminbi per cubic metre (RMB·m^-3^), according to the local charging standard. The cost of seeds, fertilizers, and pesticides was 8500 RMB·ha^-1^, and the dried root of woad was valued at the local market price of 8.20 RMB·kg^-1^. The production cost of woad in each water deficit treatment ranged from 8797.07 to 8868.81 RMB·ha^-1^, and the output value ranged from 56642.21 to 69482.32 RMB·ha^-1^ (**Table 3**). The WT1 treatment showed the highest output value and net income; the latter was increased by 1.08% compared with CK. The net income of WT4 was 59,337.60 RMB·ha^-1^, which was not higher but closer than the CK. The WT5 treatment showed the lowest net income (47831.44 RMB·ha^-1^), which was 20.27% less than the CK. The net income in other water deficit treatments was 7.69–20.14% lower than that of CK. WT1 reduced the production cost without reducing the yield, thus increasing the net income. On the other hand, the WT2, WT3, WT5, and WT6 treatments reduced production cost as well as plant yield; however, the reduction in output value was greater than that in production cost, resulting in a substantial reduction in the economic benefits of woad.

**Table 3:** Economic benefits of *I. indigotica* in different water deficit treatments.

Year	Treatment	Cost of irrigation (RMB·ha^-1^)	Cost of seed, fertilizer, pesticide, etc. (RMB·ha^-1^)	Production cost (RMB·ha^-1^)	Yield (kg·ha^-1^)	Output value (RMB·ha^-1^)	Net income (RMB·ha^-1^)	Output/input ratio
2017	WT1	333.66b	8500.00	8833.66b	8390.80a	68804.56a	59970.90a	7.79a
	WT2	324.57b	8500.00	8824.57bc	7462.24b	61190.37b	52365.80b	6.93c
	WT3	286.70d	8500.00	8786.70c	6800.36c	55762.95c	46976.25c	6.35d
	WT4	330.75b	8500.00	8830.75b	8235.32a	67529.62a	58698.87a	7.65a
	WT5	294.79c	8500.00	8794.79c	6819.79c	55922.28c	47127.48c	6.36d
	WT6	331.16b	8500.00	8831.16b	7713.45b	63250.29b	54419.13b	7.16 b
	CK	358.61a	8500.00	8858.61a	8322.25a	68242.45a	59383.84a	7.70a
2018	WT1	332.05b	8500.00	8832.05b	8475.38a	69498.12a	60666.06a	7.87a
	WT2	327.39b	8500.00	8827.39b	7638.14b	62632.75b	53805.36b	7.10b
	WT3	288.96d	8500.00	8788.96c	6986.12c	57286.18c	48497.23c	6.52c
	WT4	329.43b	8500.00	8829.43b	8308.44a	68129.21a	59299.78a	7.72a
	WT5	298.68c	8500.00	8798.68c	6923.72c	56774.50c	47975.83c	6.45c
	WT6	324.09 b	8500.00	8824.09b	7846.42b	64340.64b	55516.55b	7.29b
	CK	360.78a	8500.00	8860.78a	8348.91a	68461.06a	59600.28a	7.73a
2019	WT1	369.96b	8500.00	8869.96b	8554.18a	70144.28a	61274.31a	7.91a
	WT2	353.71c	8500.00	8853.71c	7623.76c	62514.83c	53661.12c	7.06c
	WT3	315.54e	8500.00	8815.54e	6959.82d	57070.52d	48254.98d	6.47d
	WT4	355.21c	8500.00	8855.21c	8398.70a	68869.34a	60014.13a	7.78a
	WT5	338.85d	8500.00	8838.85d	6979.25d	57229.85d	48391.00d	6.47d
	WT6	372.20b	8500.00	8872.20b	7934.63b	65063.97b	56191.77b	7.33b
	CK	387.02a	8500.00	8887.02a	8521.77a	69878.51a	60991.49a	7.86a
Average	WT1	345.23ab	8500.00	8845.23ab	8473.45a	69482.32a	60637.09a	7.86a
	WT2	335.22ab	8500.00	8835.22ab	7574.71c	62112.65c	53277.43c	7.03b
	WT3	297.07c	8500.00	8797.07c	6915.43d	56706.55d	47909.49d	6.45c
	WT4	338.46ab	8500.00	8838.46ab	8314.15a	68176.06a	59337.60a	7.71a
	WT5	310.77bc	8500.00	8810.77bc	6907.59d	56642.21d	47831.44d	6.43c
	WT6	342.48ab	8500.00	8842.48ab	7831.50b	64218.30b	55375.82b	7.26b
	CK	368.81a	8500.00	8868.81a	8397.64a	68860.68a	59991.87a	7.76a

Different lowercase letters in the same column indicate significant differences (P< 0.05).

### 3.5 I. indigotica quality

#### 3.5.1 Indigo content

**Figure 11:**
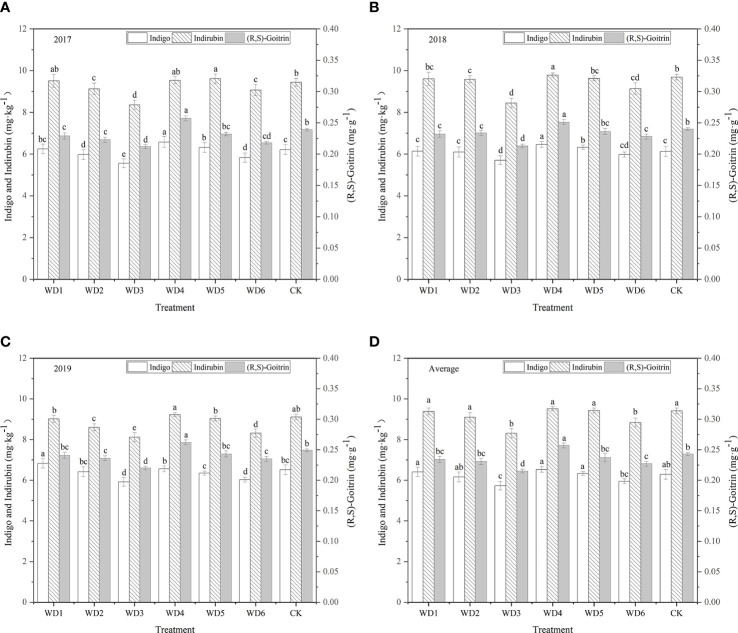
Indigo, indirubin, and (R,S)-goitrin contents of *I. indigotica* plants in different water deficit treatments. **(A–D)** describe the indigo, indirubin, and (R,S)-goitrin contents in 2017, 2018, 2019, and the average of these three years, respectively. Different lowercase letters in the same column indicate significant differences (*P*< 0.05). Data were presented as mean ± SE (n = 3).

**Figure 11** shows the indigo contents of plants were higher in WT1 and WT4 treatments than in CK. The highest indigo content (6.53 mg·kg^-1^) was found in WT4, which was 3.82% higher than CK, and the corresponding value in WT1 was 6.41 mg·kg^-1^, and there was no significant difference (*P* > 0.05) among WT1, WT4, and CK. Compared with CK, the WT6 treatment resulted in a 5.41% reduction in indigo content, and WT3 resulted in a significant (8.90%) reduction in indigo content. These results indicate that mild water deficit decreased the indigo content of woad during vegetative growth and fleshy root maturity, whereas severe water deficit significantly decreased the indigo content of woad.

#### 3.5.2 Indirubin content

As shown in **Figure 11**, regulated water deficit at the different growth stages of woad had different effects on the accumulation of indirubin. The content of indirubin was the highest in WT4, which was higher than CK by 1.17% (*P* > 0.05), indicating that the accumulation of indirubin was favored by continuous mild water deficit. All treatments, except WT4 and WT5, reduced the indirubin content by 0.32–11.69%. Compared with CK, the content of indirubin in WT1 and WT2 was lower by 0.32% and 3.29%, respectively, although the difference was not significant. Compared with CK, the content of indirubin in WT3 and WT6 treatments was significantly lower (*P*< 0.05) by 11.69% and 6.06%, respectively. The lowest concentration of indirubin (8.31 mg·kg^-1^) was found in WT3, indicating that severe water deficit is unfavourable for the accumulation of indirubin in woad.

#### 3.5.3 (R,S)-goitrin content

The WT4 treatment showed the highest content of (R,S)-goitrin (0.257 mg·g^-1^), which was significantly improved by 5.76% over the CK (*P*< 0.05), indicating that continuous mild water deficit treatment promotes the accumulation of (R,S)-goitrin (**Figure 11**). All the other water deficit treatments reduced the accumulation of (R,S)-goitrin. The content of (R,S)-goitrin in WT1, WT2, and WT5 treatments was the similar as that in CK (*P* > 0.05), and the content of (R,S)-goitrin in WT3 and WT6 was significantly reduced over the CK (0.028 and 0.016 mg·g^-1^, respectively).

#### 3.5.4 Polysaccharide content

**Figure 12:**
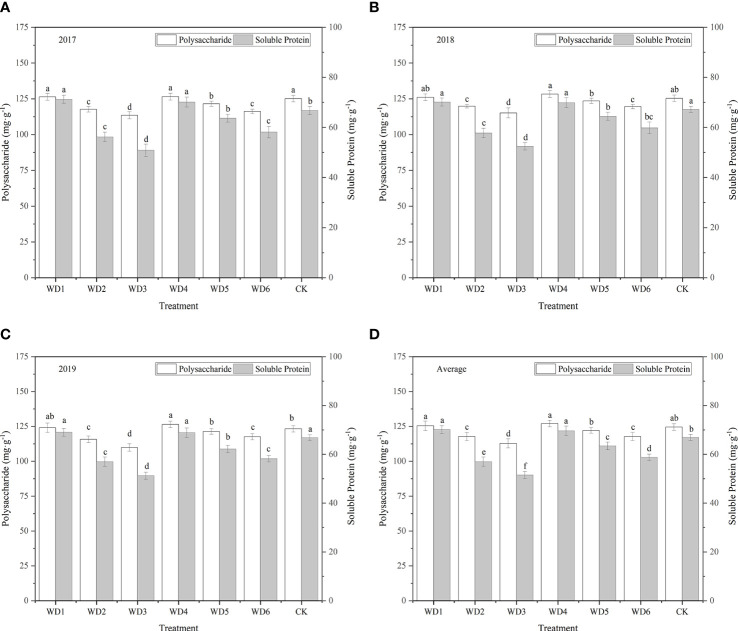
Polysaccharide and soluble protein contents of *I. indigotica* plants in different water deficit treatments. **(A–D)** describe the polysaccharide and soluble protein contents in 2017, 2018, 2019, and the average of these three years, respectively. Different lowercase letters in the same column indicate significant differences (*P*< 0.05). Data were presented as mean ± SE (n = 3).

As shown in **Figure 12**, all water deficit treatments affected the polysaccharide content of woad root. The polysaccharide content of plants was the highest in WT4 (127.09 mg·g^-1^), and was 0.73% and 2.01% higher in WT1 and WT4, respectively, than in CK. No significant difference (*P* > 0.05) was detected in the plant polysaccharide content among WT1, WT4, and CK. All other water deficit decreased the polysaccharide content of woad roots by 1.94–9.43% compared with CK. The polysaccharide content of plants in WT5 was similar to that in CK. Significant difference in polysaccharide content was not detected between WT2 and WT6 treatments. However, polysaccharide content in WT2 and WT6 was significantly reduced by 5.47% and 5.44%, respectively, compared with CK. The WT3 treatment showed the greatest decrease in polysaccharide content (9.43%) compared with CK. The results showed that mild water deficit was conducive to the accumulation of polysaccharides during vegetative growth and fleshy root growth, while moderate and severe water deficit during vegetative growth and fleshy root growth and mild water deficit during fleshy root maturity were unfavorable to the formation of polysaccharides.

#### 3.5.5 Soluble protein content

As shown in **Figure 12**, variation in the content of soluble proteins in woad root in different treatments was close to that in the content of polysaccharides. The soluble protein contents in WT1 and WT4 were 70.12 and 69.60 mg·g^-1^, respectively, which were significantly increased (*P*< 0.05) over the CK by 4.80% and 4.02%, respectively. The soluble protein contents in all the other water deficit treatments were significantly less than that in CK by 5.2–23.0%. The soluble protein content was the lowest in WT3 (51.52 mg·g^-1^), which was 23.0% less than that in CK. These findings indicate that mild water deficit during vegetative growth and fleshy root growth enhances the soluble proteins accumulation, while moderate and severe water deficit negatively impacts the formation of soluble proteins in woad root, thus decreasing the soluble protein content of root. The soluble protein content in WT6 was significantly lower than that in WT1 by 12.24%, indicating that mild water deficit during the fleshy root maturity was not conducive to the accumulation of soluble proteins in *I. indigotica*.

## 4 Discussion

### 4.1 Crop water consumption

Application of the water deficit diminished the soil water content, inhibited transpiration from leaves, and reduced the water consumption of the crop. Well timed and properly deficit irrigation could decrease total crop water consumption and enhance WUE, without significantly reducing the yield, thus facilitating water conservation, high yield, and high efficiency. In sugar beet production (Topak et al., 2010), deficit irrigation techniques can reduce irrigation water use by 25% compared to fully irrigated techniques. In soybean, application of the I_85%_ (85% of ETc) treatment facilitated 15% irrigation water saving and provided the same yield under limited irrigation water conditions (Abd El-Wahed et al., 2017). Studies on the deficit regulation irrigation of peach trees (Wang et al., 2020) showed that deficit irrigation with up to 40% water reduction over an 8–9-year period resulted in no significant yield loss while effectively suppressing peach branch growth. Our study found that the total water consumption of *I. indigotica* (woad) was the highest (387.38 mm) under sufficient water supply (CK). Total water consumption in all other treatments was significantly lower (*P*< 0.05) than that of CK by 4.44–10.21%. The severe water deficit treatment (WT3) caused the least total water consumption (347.83 mm), which was significantly reduced by 10.21% compared to CK, indicating that total water consumption of woad decreased with the increase in deficit degree. This result is compatible with Ertek and Kara (2013), who showed that deficit irrigation reduced total water consumption in sweet corn, and increased with the aggravation of regulated deficit. In table grapes, deficit irrigation improves the quality of berries and decreases water consumption (Pinillos et al., 2016).

The water consumption of woad at each growth stage showed a single peak curve, characterized by an initial increase, followed by a decline. Water consumption was the lowest at the seedling stage, accounting for 9.02–9.65% of the total water consumption. This was mainly due to low temperature and insufficient light as well as the small size, slow growth rate, less water requirement and weak transpiration of woad plants. Water consumption was the highest during vegetative growth and fleshy root growth of woad, mainly because of the rapid growth of stems, leaves, and roots and the rapid increase in leaf area, transpiration rate, taproot length, and taproot diameter. Water consumption during the fleshy root maturity period (58.09–76.72 mm) was significantly lower than that fleshy root growth, mainly due to the lower temperature, sunlight intensity, and leaf area during the fleshy root maturity period, which decreased the transpiration of woad plants. Therefore, the water consumption of woad was influenced by the water deficit degree and stage. Similar findings have been made by previous studies (Tari, 2016), water consumption was related to the period of water deficit application.

The study showed that the daily water consumption intensity and modulus of woad at each growth stage first increased and then decreased, and showed the following order: seedling< fleshy root maturity< vegetative growth and fleshy root growth. Mild water deficit treatment (WT1 and WT4) during vegetative growth and fleshy root growth showed no significant differences (*P* > 0.05) in water consumption modulus compared with CK during vegetative growth, and the daily water consumption intensity of mild water deficit (WT1) during vegetative growth was lower by 7.20% compared with CK. Moderate (WT2 and WT5) and severe (WT3) water deficit significantly reduced the daily water consumption intensity by 12.47–22.44%, and moderate water deficit (WT5) during vegetative growth and fleshy root growth periods and severe water deficit (WT3) during vegetative growth significantly reduced the water consumption modulus by 6.24% and 9.58%, respectively, compared with CK. These results indicate that mild water deficit does not significantly reduce the water consumption intensity and modulus of woad, whereas moderate and severe water deficits do. The water consumption intensity of woad was lowest at the seedling stage and highest at vegetative and fleshy root growth, probably because of the physiological characteristics of the crop and transpiration from plants.

### 4.2 Dry matter accumulation

In general, dry matter distribution was affected by the degree of water deficit. With the increase in water deficit, more dry matter distribution was partitioned to the root system. Liu et al. (2016) found that mild water deficit treatment improves canopy architecture, while moderate water deficit treatment improves dry matter distribution, thus enhancing winter wheat yield. In this study, we found that total dry matter accumulation in mild water deficit (WT1, WT4 and WT6) was no significant difference from that in CK (*P* > 0.05), while moderate (WT2 and WT5) and severe (WT3) deficit significantly reduced total dry matter accumulation by 12.83–28.75% to CK. Dry matter accumulation in the mild water deficit treatment was higher than that in moderate and severe water deficit treatments, which suggests that unlike mild water deficit, both moderate and severe water deficits cause a significant reduction in dry matter accumulation. After rehydration during fleshy root growth, the highest total dry matter accumulation (19.78 g) was recorded under mild water deficit at vegetative growth (WT1), which was similar to the CK, because of the compensatory growth effect of woad. Severe water deficit (WT3) resulted in the lowest total dry matter accumulation (15.78 g), which was increased by 9.56 g compared with the vegetative growth period. After rehydration, more photosynthetic products were allocated to the root in moderate water deficit, which improved plant yield, whereas the severe water deficit treatment allocated more photosynthetic products to the aboveground part (Li et al., 2019). Chartzoulakis et al. (1993) found that water deficit increased the distribution of dry matter to roots, however, the total dry matter content of roots remained unchanged. This was not totally in line with our results, probably due to the fact that woad has a deep root system and therefore shows a complex response to water deficit (Fabeiro et al., 2003).

### 4.3 Yield and WUE

Different degrees and stages of water deficit affect the dry matter partitioning, which consequently affects winter wheat yield (Zhang et al., 2012). Previous studies (Sun et al., 2006; Chen et al., 2013) have shown that timely and moderate deficit irrigation or drought stress reduces total water consumption, improves WUE, and optimizes all physiological indicators of plants, without significantly affecting yield. In this study, different levels of water deficit had different effects on plant yield. Under mild water deficit at vegetative growth (WT1) and fleshy root growth (WT4) stages, the economic yield of woad was not significant different from CK; however, the WUE was significantly higher than CK by 7.84–6.92%. These results indicate that rehydration treatment after mild water deficit did not significantly decrease the economic yield and WUE of woad, because of the rehydration compensation effect produced by the rehydration treatment. This is close to the results in maize (Zou et al., 2021). The detrimental effect of water stress on yield during the early to mid-nutritional stages can be recovered by 80% or 100% irrigation in later periods.

The yield and WUE of woad were not the highest in CK, despite adequate water supply during the whole growth period. Seventy-five percent irrigation (45 mm) treatment increased the WUE of wheat plants at all growth stages, whereas the control treatment (full irrigation) achieved a lower WUE (Meena et al., 2019). This may be because under conditions of adequate water supply, a larger canopy structure leads to poorer aeration capacity, which consequently reduces soil oxygen concentration, root activity, and the allocation of photosynthetic products to the root system. Severe water stress resulted in excess nutrient transfer to winter wheat seeds, causing faster water loss (Yan et al., 2019). Compared with CK, the economic yield and WUE of woad in moderate (WT2 and WT5) and severe (WT3) water deficit was significantly lower by 9.80–17.74% and 5.63–10.24%, respectively, indicating that both moderate and severe water deficits significantly reduce the economic yield and WUE of woad. This is consistent with the results of Patane et al. (2011) on processed tomatoes. Hazrati et al. (2017) also showed similar results; severe water deficit treatments limited plant growth and significantly reduced yield. The economic yield and WUE of woad under mild water deficit during fleshy root maturity (WT6) were significantly lower than those under mild water deficit during other stages, illustrating that the economic yield and WUE of woad were also impacted by the period of water deficit.

### 4.5 Quality of *I. indigotica*

Many external factors affected the quality of woad, including plant variety and origin, meteorological factors, cultivation method, soil moisture content, and soil ecological environment. The accumulation of primary and secondary metabolites determines the yield and quality, respectively. Our results found that continuous mild water deficit (WT4) resulted in the highest contents of indigo and indirubin (6.53 mg·kg^-1^ and 9.52 mg·kg^-1^), respectively, which were 3.82% and 1.17%, respectively, higher than the values in CK, indicating that continuous mild water deficit promotes the accumulation of the active ingredients indigo and indirubin. The content of (R,S)-goitrin under continuous mild water deficit (WT4) was significantly higher than that under CK conditions by 5.76%, while the other water deficit treatments reduced (R,S)-goitrin content by 2.47–11.52%. These findings illustrate that mild water deficit treatment favors the accumulation of (R,S)-goitrin, while moderate and severe water deficits do not. This is in accordance with the results of Wang Z. Y. et al. (2021). Continuous mild and moderate water deficit increased the content of (R,S)-goitrin, indirubin, and indigo, whereas severe water deficit was unfavorable to the accumulation of the active components of woad (Wang Z. Y. et al., 2021). Under tolerable drought stress conditions, large amounts of photosynthetic products accumulate in the plant, and the “excess” amount of photosynthetic products is converted into carbon-containing secondary metabolites by the plant, increasing the content of secondary metabolites for sustenance. Compared with mild water deficit during vegetative and fleshy root growth (WT4), the contents of indigo, indirubin, and (R,S)-goitrin were significantly decreased by 8.88%, 7.14%, and 11.67%, respectively, under mild water deficit at vegetative growth and fleshy root maturity (WT6), indicating that these active ingredients contents under the above-described conditions were not only affected by the water deficit degree but also related to water deficit period.

Ordinarily, the enhancement of fruit quality by water stress is accompanied by a reduction in yield (Ozbahce and Tari, 2010; Ripoll et al., 2014; Candido et al., 2015; Lovelli et al., 2017). Ballester et al. (2011) concluded that regulated deficit irrigation (RDI) increased the total soluble solid (TSS) content, titratable acidity, and fruit quality of citrus trees. Wang et al. demonstrated that the TSS content, vitamin C, and reducing sugar content were higher in tomato fruits after water deficit compared with the control (Wang C .X. et al., 2015a; Wang X. P. et al., 2015). In this study, we found that the polysaccharide content of woad root under mild water deficit at vegetative (WT1) and fleshy root growth (WT4) stages increased by 0.73% and 2.01%, respectively, compared to CK, whereas other water deficit decreased the polysaccharide content by 1.94–9.43%. The soluble protein content of woad root under mild water deficit (WT1 and WT4) at vegetative and fleshy root growth was 70.12 and 69.60 mg·g^-1^, respectively, which was significantly higher than that in CK by 4.80% and 4.02%, respectively. The soluble protein content in other water deficit was significantly less than that in CK by 5.20–23.00%. These results indicate that mild water deficit promotes the accumulation of polysaccharides and soluble proteins, whereas moderate and severe water deficit do not. In another study, the single plant yield of tomato under mild water stress was better than that under adequate water supply by 18.9% and 1.0% in 2016 and 2017, respectively, and mild drought improved fruit quality parameters (fruit hardness, fruit color index, TSS content, and total soluble sugar concentration) (Yang et al., 2019).

## 5 Conclusion

Total water consumption in water deficit was significantly less than that in the control, and the decline in water consumption increased with the aggravation of water deficit. Total dry matter accumulation in moderate (WT2 and WT5) and severe water deficit (WT3) was significantly less than that in the control, thereby reducing *I. indigotica* (woad) yield. The yield reduction under mild water deficit during vegetative growth (WT1) and fleshy root growth (WT4) was not significant compared with the control; however, WUE was significantly increased under mild water deficit. The accumulation of indigo, indirubin, (R,S)-goitrin, polysaccharides, and soluble proteins was favored by continuous mild water deficit (WT4), and the amounts of these active ingredients were higher under the above-mentioned conditions than in the control. In conclusion, the application of continuous mild water deficit during vegetative growth and fleshy root growth (WT4) affects dry matter accumulation and distribution by reducing water consumption, thus maintaining high yield and enhancing the quality of woad. Thus, continuous mild water deficit irrigation during vegetative growth and fleshy root growth (WT4) is the best strategy for the cultivation of woad in Hexi Oasis, China. In this study, the variation in different water deficit was considered, and fertilizer and mulching methods were not addressed. Therefore, further research is needed to understand water–fertilizer interactions and mulching regimes.

## Data availability statement

The original contributions presented in the study are included in the article/supplementary material. Further inquiries can be directed to the corresponding author.

## Author contributions

CZ prepared the experimental scheme, data analysis and drafted the article. HZ revised the experimental protocol and article format. YuW, FL, YoW, and ZW performed part of the experiments and provided some of the experimental results for the manuscript. All authors approved the final version of the manuscript.

## Funding

This work was funded by the Industrial Support Plan Project of Gansu Provincial Department of Education (No. 2022CYZC-51), the National Natural Science Foundation of China (No. 52269008), the Key Research and Planning Projects of Gansu Province (No. 18YF1NA073), and the Project of “Innovation Star” for Excellent Graduate Students in Gansu Province (No. 2022CXZX-660).

## Acknowledgments

We thank the Industrial Support Plan Project of Gansu Provincial Department of Education (No. 2022CYZC-51), the National Natural Science Foundation of China (No. 52269008), the Key Research and Planning Projects of Gansu Province (No. 18YF1NA073), and the Project of “Innovation Star” for Excellent Graduate Students in Gansu Province (No. 2022CXZX-660) for funding and laboratory facilities.

## Conflict of interest

The authors declare that the research was conducted in the absence of any commercial or financial relationships that could be construed as a potential conflict of interest.

## Publisher’s note

All claims expressed in this article are solely those of the authors and do not necessarily represent those of their affiliated organizations, or those of the publisher, the editors and the reviewers. Any product that may be evaluated in this article, or claim that may be made by its manufacturer, is not guaranteed or endorsed by the publisher.

## References

[B1] Abd El-Wahed M. H. Baker G. A. Ali M. M. Abd El-Fattah F. A. (2017). Effect of drip deficit irrigation and soil mulching on growth of common bean plant, water use efficiency and soil salinity. Sci. Hortic. 225, 235–242. doi: 10.1016/j.scienta.2017.07.007

[B2] Ballester C. Castel J. Intrigliolo D. S. Castel J. R. (2011). Response of clementina de nules citrus trees to summer deficit irrigation. yield components and fruit composition. Agric. Water Manage. 98 (6), 1027–1032. doi: 10.1016/j.agwat.2011.01.011

[B3] Bumgarner N. R. Scheerens J. C. Kleinhenz M. D. (2012). Nutritional yield: a proposed index for fresh food improvement illustrated with leafy vegetable data. Plant Food Hum. Nutr. 67 (3), 215–222. doi: 10.1007/s11130-012-0306-0 22922881

[B4] Candido V. Campanelli V. D’Addabbo T. Castronuovo D. Perniola M. Camele I. (2015). Growth and yield promoting effect of artificial mycorrhization on field tomato at different irrigation regimes. Sci. Hortic. 187, 35–43. doi: 10.1016/j.scienta.2015.02.033

[B5] Cano-Lamadrid M. Giron I. F. Pleite R. Burlo F. Corell M. Moriana A.. (2015). Quality attributes of table olives as affected by regulated deficit irrigation. Lwt Food Sci. Technol. 62 (1), 19–26. doi: 10.1016/j.lwt.2014.12.063

[B6] Carbonell-Barrachina A. A. Memmi H. Noguera-Artiaga L. Gijon-Lopez M. D. Ciapa R. Perez-Lopez D. (2015). Quality attributes of pistachio nuts as affected by rootstock and deficit irrigation. J. Sci. Food Agric. 95 (14), 2866–2873. doi: 10.1002/jsfa.7027 25428819

[B7] Chai Q. Gan Y. T. Zhao C. Xu H. L. Waskon R. M. Niu Y. N.. (2016). Regulated deficit irrigation for crop production under drought stress: a review. Agron. Sustain. Dev. 36, (1). doi: 10.1007/s13593-015-0338-6

[B8] Chartzoulakis K. Noitsakis B. Therios I. (1993). Photosynthesis, plant growth and carbon allocation in kiwi cv. Hayward, as influenced by water deficits. Acta Hortic. 335, 227–234. doi: 10.17660/ActaHortic.1993.335.26

[B9] Chen J. Kang S. Du T. Qiu R. Guo P. Chen R. (2013). Quantitative response of greenhouse tomato yield and quality to water defificit at difffferent growth stages. Agric. Water Manage. 129, 152–162. doi: 10.1016/j.agwat.2013.07.011

[B10] Cui J. Shao G. Lu J. Keabetswe L. Hoogenboom G. (2020). Yield, quality and drought sensitivity of tomato to water deficit during different growth stages. Sci. Agric. 77, (2). doi: 10.1590/1678-992x-2018-0390

[B11] Ertek A. Kara B. (2013). Yield and quality of sweet corn under deficit irrigation. Agric. Water Manage. 129, 138–144. doi: 10.1016/j.agwat.2013.07.012

[B12] Fabeiro C. Martin D. S.O.F. Lopez R. Dominguez A. (2003). Production and quality of the sugar beet (beta vulgaris, l.) cultivated under controlled deficit irrigation conditions in a semi-arid climate. Agric. Water Manage. 62, 215–227. doi: 10.1016/S0378-3774(03)00097-0

[B13] Favati A. Lovelli S. Galgano F. Miccolis V. Tommaso T. D. Candido V. (2009). Processing tomato quality as affected by irrigation scheduling. Sci. Hortic. 122, 562–571. doi: 10.1016/j.scienta.2009.06.026

[B14] Geerts S. Raes D. (2009). Deficit irrigation as an on-farm strategy to maximize crop water productivity in dry areas. Agr. Water Manage. 96 (9), 1275–1284. doi: 10.1016/j.agwat.2009.04.009

[B15] Geng G. Wu J. Wang Q. Lei T. He B. Li X.. (2016). Agricultural drought hazard analysis during 1980 - 2008: a global perspective. Int. J. Climatol. 36, 389–399. doi: 10.1002/joc.4356

[B16] Giuliani M. M. Gatta G. Nardella E. Tarantino E. (2016). Water saving strategies assessment on processing tomato cultivated in Mediterranean region. Ital. J. Agron. 11, 69–76. doi: 10.4081/ija.2016.738

[B17] Graham C. Beck R. Thavarajah D. (2018). Dietary reference intake and nutritional yield of lentils in the northern great plains. Crop Sci. 58 (3), 1342–1348. doi: 10.2135/cropsci2017.10.0617

[B18] Han J. Jiang X. Zhang L. (2011). Optimisation of extraction conditions for polysaccharides from the roots of isatis tinctoria l. by response surface methodology and their *in vitro* free radicals scavenging activities and effects on IL-4 and IFN-γ mRNA expression in chicken lymphocytes. Carbohydr. Polym. 86, 1320–1326. doi: 10.1016/j.carbpol.2011.06.036

[B19] Hazrati S. Tahmasebi-Sarvestani Z. Mokhtassi-Bidgoli A. Mohammadi H. Nicola S. (2017). Effects of zeolite and water stress on growth, yield and chemical compositions of aloe vera l. Agric. Water Manage. 181, 66–72. doi: 10.1016/j.agwat.2016.11.026

[B20] Kang S. Z. Hao X. M. Du T. S. Tong L. Su X. L. Lu H. N.. (2017). Improving agricultural water productivity to ensure food security in China under changing environment: from research to practice. Agric. Water Manage. 179, 5–17. doi: 10.1016/j.agwat.2016.05.007

[B21] Ke L. Wen T. Bradshaw J. P. Zhou J. Rao P. (2012). Antiviral decoction of isatidis radix (b n lán gKn) inhibited a influenza virus adsorption on MDCK cells by cytoprotective activity. J. Tradit. Complement. Med. 2, 47–51. doi: 10.1016/s2225-411030070-0 24716114 PMC3943010

[B22] Kusçu H. Turha A. Demir A. O. (2014). The response of processing tomato to deficit irrigation at various phenological stages in a subhumid environment. Agric. Water Manage. 133, 92–103. doi: 10.1016/j.agwat.2013.11.008

[B23] Lahoz I. Perez-de-Castro A. Valcarcel M. Macua J. I. Beltran J. Rosello S.. (2016). Effect of water deficit on the agronomical performance and quality of processing tomato. Sci. Hortic. 200, 55–65. doi: 10.1016/j.scienta.2015.12.051

[B24] Li Y. Y. Liu N. N. Fan H. Su J. X. Fei C. Wang K. Y.. (2019). Effects of deficit irrigation on photosynthesis, photosynthate allocation, and water use efficiency of sugar beet. Agric. Water Manage 223, 105701. doi: 10.1016/j.agwat.2019.105701

[B25] Liu E. K. Mei X. R. Yan C. R. Gong D. Z. Zhang Y. Q. (2016). Effects of water stress on photosynthetic characteristics, dry matter translocation and WUE in two winter wheat genotypes. Agric. Water Manage. 167, 75–85. doi: 10.1016/j.agwat.2015.12.026

[B26] Liu X. Qi Y. Li F. Yang Q. Yu L. (2018). Impacts of regulated deficit irrigation on yield, quality and water use efficiency of arabica coffee under different shading levels in dry and hot regions of southwest China. Agric. Water Manage. 204, 292–300. doi: 10.1016/j.agwat.2018.04.024

[B27] Liu F. L. Shahnazari A. Andersen M. N. Jacobsen S. E. Jensen C. R. (2006). Physiological responses of potato (Solanum tuberosum l.) to partial root-zone drying: ABA signalling, leaf gas exchange, and water use efficiency. J. Exp. Bot. 57, 3727–3735. doi: 10.1093/jxb/erl131 16982651

[B28] Lovelli S. Potenza G. Castronuovo D. Perniola M. Candido V. (2017). Yield, quality and water use efficiency of processing tomatoes produced under different irrigation regimes in Mediterranean environment. Ital. J. Agron. 12, 17–24. doi: 10.4081/ija.2016.795

[B29] Mckenzie F. C. Williams J. (2015). Sustainable food production: constraints, challenges and choices by 2050. Food Secur. 7, 221–233. doi: 10.1007/s12571-015-0441-1

[B30] Meena R. P. Karnam V. Tripathi S. C. Jha A. Sharma R. K. Singh G. P. (2019). Irrigation management strategies in wheat for efficient water use in the regions of depleting water resources. Agric. Water Manage. 214, 38–46. doi: 10.1016/j.agwat.2019.01.001

[B31] Meng X. Zhang S. Zhang Y. (2013). Temporal and spatial changes of temperature and precipitation in hexi corridor during 1955–2011. J. Geogr. Sci. 23, 653–667. doi: 10.1007/s11442-013-1035-5

[B32] Nyathi M. K. Van Halsema G. E. Beletse Y. G. Annandale J. G. Struik P. C. (2018). Nutritional water productivity of selected leafy vegetables. Agric. Water Manage. 209, 111–122. doi: 10.1016/j.agwat.2018.07.025

[B33] Ozbahce A. Tari A. F. (2010). Effects of emitter space and water stress on yield and quality of processing tomato under semi-arid climate conditions. Agric. Water Manage. 97, 1405–1410. doi: 10.1016/j.agwat.2010.04.008

[B34] Patane C. Tringali S. Sortino O. (2011). Effects of deficit irrigation on biomass, yield, water productivity and fruit quality of processing tomato under semi-arid Mediterranean climate conditions. Sci. Hortic. 129, 590–596. doi: 10.1016/j.scienta.2011.04.030

[B35] Pelling M. Maskrey A. Ruiz P. Hall P. Peduzzi P. Dao Q.H. (2004). Reducing Disaster Risk: A Challenge for Development. United Nations, New York

[B36] Pinillos V. Chiamolera F. M. Ortiz J. F. Hueso J. J. Cuevas J. (2016). Post-veraison regulated deficit irrigation in ‘Crimson seedless’ table grape saves water and improves berry skin color. Agric. Water Manage. 165, 181–189. doi: 10.1016/j.agwat.2015.11.007

[B37] Ripoll J. Urban L. Staudt M. Lopez-Lauri F. Bidel L. P. R. Bertin N. (2014). Water shortage and quality of fleshy fruits-making the most of the unavoidable. J. Exp. Bot. 65, 4097–4117. doi: 10.1093/jxb/eru197 24821951

[B38] Saleh M. I. Kiyoshi O. Nur A. K. (2008). Influence of single and multiple water application timings on yield and water use efficiency in tomato (var. first power). Agric. Water Manage. 95, 116–122. doi: 10.1016/j.agwat.2007.09.006

[B39] Shahnazari A. Liu F. Andersen M. N. Jacobsen S. E. Jensen C. R. (2007). Effects of partial root-zone drying on yield, tuber size and water use efficiency in potato under field conditions. Field Crops Res. 100 (1), 117–124. doi: 10.1016/j.fcr.2006.05.010

[B40] Shahzad A. Xu Y. Ma X. Irshad A. Muhammad K. Dong Z.. (2017). Planting patterns and deficit irrigation strategies to improve wheat production and water use efficiency under simulated rainfall conditions. Front. Plant Sci. 8. doi: 10.3389/fpls.2017.01408 PMC5572266 28878787

[B41] Sun H. Y. Liu C. M. Zhang X. Y. Shen Y. J. Zhang Y. Q. (2006). Effects of irrigation on water balance, yield and WUE of winter wheat in the north China plain. Agric. Water Manage. 85, 211–218. doi: 10.1016/j.agwat.2006.04.008

[B42] Tari A. F. (2016). The effects of different deficit irrigation strategies on yield, quality, and water-use efficiencies of wheat under semi-arid conditions. Agric. Water Manage. 167, 1–10. doi: 10.1016/j.agwat.2015.12.023

[B43] Topak R. Suheri S. Acar B. (2010). Comparison of energy of irrigation regimes in sugar beet production in a semi-arid region. Energy 35 (12), 5464–5471. doi: 10.1016/j.energy.2010.06.018

[B44] Wang C. X. Gu F. Chen J. L. Yang H. Jiang J. J. Du T. S.. (2015). Assessing the response of yield and comprehensive fruit quality of tomato grown in greenhouse to deficit irrigation and nitrogen application strategies. Agric. Water Manage. 161, 9–19. doi: 10.1016/j.agwat.2015.07.010

[B45] Wang Y. C. He X. C. Li F. Q. Deng H. L. Wang Z. Y. Huang C. X.. (2021). Effects of water and nitrogen coupling on the photosynthetic characteristics, yield, and quality of isatis indigotica. Sci. Rep UK. 11 (1), 17356. doi: 10.1038/s41598-021-96747-0 PMC8405819 34462495

[B46] Wang X. P. Huang G. H. Yang J. S. Huang Q. Z. Liu H. J. Yu L. P. (2015). An assessment of irrigation practices: sprinkler irrigation of winter wheat in the north China plain. Agric. Water Manage. 159, 197–208. doi: 10.1016/j.agwat.2015.06.011

[B47] Wang D. Zhang H. H. Gartung J. (2020). Long-term productivity of early season peach trees under different irrigation methods and postharvest deficit irrigation. Agric. Water Manage 230, 105940. doi: 10.1016/j.agwat.2019.105940

[B48] Wang Z. Y. Zhang H. J. Wang Y. C. Zhou C. L. (2021). Integrated evaluation of the water deficit irrigation scheme of indigowoad root under mulched drip irrigation in arid regions of Northwest China based on the improved TOPSIS method. Water 13, (11). doi: 10.3390/w13111532

[B49] Win S. K. Zamora O. B. Thein S. (2014). Determination of the water requirement and kc values of sugarcane at different crop growth stages by lysimetric method. Sugar Tech. 16 (3), 286–294. doi: 10.1007/s12355-013-0282-1

[B50] Yang H. Du T. S. Mao X. M. Ding R. S. Shukla M. K. (2019). A comprehensive method of evaluating the impact of drought and salt stress on tomato growth and fruit quality based on EPIC growth model. Agric. Water Manage. 213, 116–127. doi: 10.1016/j.agwat.2018.10.010

[B51] Yan S. C. Wu Y. Fan J. L. Zhang F. C. Qiang S. C. Zheng J.. (2019). Effects of water and fertilizer management on grain filling characteristics, grain weight and productivity of drip-fertigated winter wheat. Agric. Water Manage. 213, 983–995. doi: 10.1016/j.agwat.2018.12.019

[B52] Yunusa I. A. M. Zerihun A. Gibberd M. R. (2018). Analysis of the nexus between population, water resources and global food security highlights significance of governance and research investments and policy priorities. J. Sci. Food Agric. 98, 5764–5775. doi: 10.1002/jsfa.9126 29749117

[B53] Zhang D. D. Du K. Zhao Y. T. Shi S. S. Wu Y. C. Jia Q.. (2019). Indole alkaloid glycosides from isatis tinctoria roots. Nat. Prod. Res. 35 (2), 244–250. doi: 10.1080/14786419.2019.1624960 31174427

[B54] Zhang Y. Q. Kang S. Z. Ward E. J. Ding R. S. Zhang X. Zheng R. (2011). Evapotranspiration components determined by sap flow and microlysimetry techniques of a vineyard in northwest China: dynamics and influential factors. Agric. Water Manage. 98, 1207–1214. doi: 10.1016/j.agwat.2011.03.006

[B55] Zhang H. P. Turner N. C. Poole M. L. (2012). Increasing the harvest index of wheat in the high rainfall zones of southern Australia. Field Crop Res. 129, 111–123. doi: 10.1016/j.fcr.2012.02.002

[B56] Zhang Z. Zhang Y. Sun Z. Zheng J. Liu E. Feng L.. (2019). Plastic film cover during the fallow season preceding sowing increases yield and water use efficiency of rain-fed spring maize in a semi-arid climate. Agric. Water Manage. 212, 203–210. doi: 10.1016/j.agwat.2018.09.001

[B57] Zong R. Wang Z. Zhang J. Li W. (2021). The response of photosynthetic capacity and yield of cotton to various mulching practices under drip irrigation in Northwest China. Agric. Water Manage 249, 106814. doi: 10.1016/j.agwat.2021.106814

[B58] Zou Y. F. Saddique Q. Ali A. Xu J. Khan M. I. Qing M.. (2021). Deficit irrigation improves maize yield and water use efficiency in a semi-arid environment. Agric. Water Manage 243, 106483. doi: 10.1016/j.agwat.2020.106483

